# Monthly Summary

**Published:** 1869-07

**Authors:** 


### MONTHLY SUMMARY.

---

*Imperfect Enamel.*—Dr. S. P. CUTLER says: Furrowed enamel is not always an indication of bad taint in blood of an hereditary nature. Not long since a young lady called on me to have her teeth examined. There were some few decays, with this exception all of her teeth were perfect, except the lower front and lateral in cisors of the left side, which were both cross-furrowed deep into the enamel, some three or four grooves in each, and otherwise not well developed. She informed me that two corresponding deciduous teeth were knocked out when she was about four years old. high respect. This statement settled the rising suspicions in my mind. The ability of the party should of itself have done away with all doubts, even without any knowledge of the case.

The above case goes to establish beyond any reasonable doubt the connection existing between the first and second sets of corresponding teeth, showing the importance of retaining the deciduous teeth up to the eruptive stage of their respective successors, and that too, in a healthy condition.

The above case also shows conclusively that the nutrition of the crowns, especially the enamel, was disturbed to a considerable extent by the accidental loss of the deciduous teeth at too early a stage ; had they been retained two years longer, no defects could have existed in the two permanent teeth referred to.

The importance of not only retaining the deciduous teeth up to the full period when nature makes provision for their leaving, by root absorption, as well as their healthy preservation, cannot be too highly estimated.—*Dental Register.*

---

*Arkansas Stones.*—Oil or Ouichita stone is the material from which are manufactured the oil stones used for giving a fine edge to edge tools. This stone is fouud in Arkansas, the quarries being situated near the celebrated Hot Springs of that State. The stone is quarried with great care into blocks of from two to four feet square, or of irregular shape, according as it lies in the quarry. From the Hot Springs it is shipped to Little Rock, where, at the present time, it is sold at the rate of three cents per pound in the rough, the purchaser being charged with all the expenses of its shipment from that place.

The white stone comes from the same quarries as the oil stone, but from a different vein. This stone is much more costly, and of a much finer grain than the oil stone. It is used by jewellers, engravers, and manufacturers of surgical instruments, used and manufactured by them. It is also used for sharpening sewing machine needles, and all delicately pointed instruments, for sharpening the instruments it is better and is much more costly than the oil stone. We believe that the quarries at the Hot Springs are the only ones producing the oil and white stone in America, and they have proved an immense fortune to the proprietors.

There are in America but five manufactories of oil and white stones. One is at Jeffersonville, two at New York, one at St. Louis, and one at New Albany. The manufactory in this city is more extensive than all other four combined, and purchases more stone and turns out more product than the other four put together.

Its annual product, is of Ouichita or oil stones, one hundred and five thousand pounds, and of white stones ten thousand pounds, and Hindostan stones one hundred and eighty thousand pounds. The value of this product is immense. The Hindostan stone comes from a quarry in Orange County, Indiana. To give an idea of the value of white stone, we will state that we are informed that seven thousand of the sewing machine whetstones were recently shipped to Albany N. Y., in a little box eight inches long, eight inches wide, and eight inches deep, for which $70 per thousand were paid, or $490 for the contents of this one little box.—*Sc. American.*

---

*Strength of Carbolic Acid Solutions.*—In view of the fact that carbolic acid is now largely in use in medicine, with a probability that its range of application will be increased, it is well for prescribers to be very careful of the particular preparation they employ. Instances are reported where much damage has been done by the external application of this substance in solution, the prescriber not knowing the exact strength of the solution, and we ourselves have seen carbolic acid ordered from the apothecaries, in such a way as to evince plainly the fact of a most blissfnl ignorance of whether the medicine was solid or a fluid, or in what proportion it was proper to use it. Dr. W. T. Channing, of Providence, reports to the Boston *Journal of Chemistry* several cases of serious results, from the use of the concentrated fluid acid, which is dispensed by some under the name of "solution carbolic acid," when the prescribers intended only a milder solution, which they had been in the habit of using, but had obtained it from other druggists. Until, therefore, some distinctive nomenclature shall be given to the various preparations of this substance, and some officinal "solution" shall be decided upon, physicians cannot be too careful in learning the strength of the solution employed, and it would be advisable to give explicit directions where to procure it.—*Med. & Surg. Reporter.*

---

*Antidote to Carbolic Acid.*—Dr. Crace Calvert states that in poisoning with this acid, the best antidote, after the stomach pump, is large doses of olive or almond oil, with a little castor oil. Oil is a solvent, and consequently a diluent of carbolic acid, and may be used to stop the corrosive effect of the acid when its action on the skin is too violent.—*Journal of Cutaneous Medicine.*

---

